# *Leishmania donovani* Develops Resistance to Drug Combinations

**DOI:** 10.1371/journal.pntd.0001974

**Published:** 2012-12-20

**Authors:** Raquel García-Hernández, José Ignacio Manzano, Santiago Castanys, Francisco Gamarro

**Affiliations:** Instituto de Parasitología y Biomedicina “López-Neyra”, IPBLN-CSIC, Parque Tecnológico de Ciencias de la Salud, Granada, Spain; University of Antwerp, Belgium

## Abstract

Drug combinations for the treatment of leishmaniasis represent a promising and challenging chemotherapeutic strategy that has recently been implemented in different endemic areas. However, the vast majority of studies undertaken to date have ignored the potential risk that *Leishmania* parasites could develop resistance to the different drugs used in such combinations. As a result, this study was designed to elucidate the ability of *Leishmania donovani* to develop experimental resistance to anti-leishmanial drug combinations. The induction of resistance to amphotericin B/miltefosine, amphotericin B/paromomycin, amphotericin B/Sb^III^, miltefosine/paromomycin, and Sb^III^/paromomycin was determined using a step-wise adaptation process to increasing drug concentrations. Intracellular amastigotes resistant to these drug combinations were obtained from resistant *L. donovani* promastigote forms, and the thiol and ATP levels and the mitochondrial membrane potential of the resistant lines were analysed. Resistance to drug combinations was obtained after 10 weeks and remained in the intracellular amastigotes. Additionally, this resistance proved to be unstable. More importantly, we observed that promastigotes/amastigotes resistant to one drug combination showed a marked cross-resistant profile to other anti-leishmanial drugs. Additionally, the thiol levels increased in resistant lines that remained protected against the drug-induced loss of ATP and mitochondrial membrane potential. We have therefore demonstrated that different resistance patterns can be obtained in *L. donovani* depending upon the drug combinations used. Resistance to the combinations miltefosine/paromomycin and Sb^III^/paromomycin is easily obtained experimentally. These results have been validated in intracellular amastigotes, and have important relevance for ensuring the long-term efficacy of drug combinations.

## Author Summary

*Leishmania* is a protozoan parasite that infects human macrophages to produce the neglected tropical disease known as leishmaniasis. Chemotherapy is currently the only treatment option for leishmaniasis. First-line therapies include pentavalent antimonials, except in some regions in the Indian subcontinent, the liposomal formulation of amphotericin B, miltefosine and paromomycin. The WHO has recently recommended a combined therapy in order to extend the life expectancy of these compounds. However, resistance could be induced in *Leishmania* if this approach is not applied in a controlled and regulated way, thus resulting in a rapid loss of efficacy of not one but two therapeutic options. In light of this, we have designed relevant experimental studies in order to determine whether *Leishmania* parasites are able to develop resistance to the different potential anti-leishmanial drug combinations that will be used in the near future. The results obtained could help us to predict the success of drug combination therapy. Experimental resistance of *Leishmania donovani* promastigotes to drug combinations was obtained after 10 weeks and remained in the intracellular amastigotes. We therefore conclude that *L. donovani* can easily develop resistance to drug combinations mainly miltefosine/paromomycin and Sb^III^/paromomycin. These results have been validated in intracellular amastigotes and are of considerable interest for future prediction of the success of drug combination therapy.

## Introduction

The use of drug combinations, either in co-formulations or co-administrations, is an established approach for the treatment of several infectious diseases including malaria and tuberculosis [1]. This approach has also recently become a priority for other tropical parasitic diseases, such as visceral leishmaniasis [2]–[6]. Leishmaniasis, a neglected tropical parasitic disease that is prevalent in 98 countries spread across three continents, is caused by protozoan parasites belonging to the genus *Leishmania* [7]. The estimated incidence of leishmaniasis is 0.2–0.4 million cases of the visceral form (VL) and 0.7–1.2 million cases of the cutaneous form (CL) [7]. Although chemotherapy is the only current treatment option for leishmaniasis, its efficacy is increasingly limited by growing resistance to first-line drugs, especially antimonials, the frequent side-effects associated with their use, and the high cost of treatment [7], [8]. The recommended first-line therapies for VL include: i) pentavalent antimonials (meglumine antimoniate and sodium stibogluconate), except in some regions in the Indian subcontinent where there are significant areas of drug resistance [9]; ii) the polyene antibiotic amphotericin B (AmB); iii) the liposomal formulation AmBisome; iv) the aminoglycoside paromomycin (PMM); and v) the oral drug miltefosine (MLF). Recently, the WHO [7], [10], recommended to use either a single dose of AmBisome or combinations of anti-leishmanial drugs in order to reduce the duration and toxicity of treatment, prolong the therapeutic life span of existing drugs and delay the emergence of resistance.

Although recent clinical trials have highlighted the efficacy and safety of anti-leishmanial drug combinations [4], [5], [10]–[12], additional clinical studies are needed to investigate various other factors, such as the identification of an effective, well-tolerated and short treatment regimen, logistical aspects, and the potential risk of developing resistance considering that compliance in field conditions can be low [13].

Herein we describe the selection and characterization of experimental resistance to drug combinations in *Leishmania* parasites. Our findings clearly demonstrate the acquisition of resistance to different drug combinations in *Leishmania donovani* promastigotes using a step-wise adaptation process to increasing drug concentrations. Similarly, and perhaps importantly, we have obtained intracellular *L. donovani* amastigotes that are resistant to different drug combinations from promastigote forms resistant to these same combinations. These results indicate different patterns of resistance depending on the drug combinations used, with the combination MLF/PMM selecting resistant *L. donovani* more rapidly than the combination AmB/PMM. Significantly, we have also observed that promastigotes/amastigotes resistant to one drug combination show a marked cross-resistance profile to other anti-leishmanial drugs, a finding that could be of major clinical relevance. Additionally, our results indicate that the resistant lines remain protected against the drug-induced loss of ATP and mitochondrial membrane potential.

## Materials and Methods

### Chemicals

Trivalent antimony (Sb^III^), paromomycin (PMM), amphotericin B (AmB), paraformaldehyde, MTT [3-(4,5-dimethylthiazol-2-yl)-2,5-diphenyltetrazolium bromide], Rhodamine 123 (Rh123), buthionine sulfoximine (BSO), FCCP (carbonyl cyanide 4-trifluoromethoxyphenylhydrazone), and Triton X-100 were obtained from Sigma-Aldrich (St. Louis, MO). Miltefosine (MLF) was purchased from Zentaris GmbH (Frankfurt am Main, Germany), and CellTiter-Glo, CellTracker, and 4′, 6-diamidino-2-phenylindole dihydrochloride (DAPI) were purchased from Invitrogen. L-glutamine and penicillin/streptomycin were obtained from Gibco. All chemicals were of the highest quality available.

### *Leishmania* culture conditions

The *L. donovani* promastigotes (MHOM/ET/67/HU3) and derivative lines used in this study were grown at 28°C in RPMI 1640-modified medium (Invitrogen) supplemented with 20% or 10% heat-inactivated fetal bovine serum (HIFBS, Invitrogen). For thiol assays, they were grown in M-199 medium (Gibco) supplemented with 10% HIFBS.

### Development of drug-resistant *Leishmania* promastigotes

The resistant lines were obtained following a previously described step-wise adaptation process [14], [15]. This process started with drug pressure in the wild-type (WT) *L. donovani* line at a concentration below the drug EC_50_ (the concentration of the drug required to inhibit parasite growth by 50%), gradually increasing the drug pressure over 10 weeks. After this period, the resistant lines were maintained for eight further weeks at the final drug concentration. The drug combination resistant lines generated, based on WHO recommendations [7], were AmB+MLF (AM), AmB+PMM (AP), AmB+Sb^III^ (the antimonial active form; AS), MLF+PMM (MP) and Sb^III^+PMM (SP). Singly resistant lines named A, M, P, and S were obtained in a similar manner. All resistant lines were maintained in the continuous presence of drugs. Resistance stability was checked at one and four months after removal from drug pressure. The EC_50_, resistance index (EC_50_ ratio for resistant and WT parasites), and cross-resistance profile were determined for each line using an MTT colorimetric assay after incubation for 72 h at 28°C in the presence of increasing concentrations of the drug, as described previously [16].

### Animals

Six-week-old male BALB/c mice were purchased from Charles River Breeding Laboratories and maintained in the Animal Facility Service of our Institute under pathogen-free conditions. They were fed a regular rodent diet and given drinking water *ad libitum*. These mice were used to collect primary peritoneal macrophages.

### Ethics statement

All experiments were performed according to National/EU guidelines regarding the care and use of laboratory animals in research. Approval for these studies was obtained from the Ethics Committee of the Spanish National Research Council (CSIC file CEA-213-1-11).

### Drug sensitivity in intracellular *Leishmania* amastigotes

Mouse peritoneal macrophages were obtained as described previously [17] and plated at a density of 1×10^5^ macrophages/well in RPMI 1640 medium supplemented with 10% HIFBS, 2 mM glutamate, penicillin (100 U/mL) and streptomycin (100 µg/mL) in 24-well tissue culture chamber slides. Late-stage promastigotes from WT and resistant lines were used to infect macrophages at a macrophage/parasite ratio of 1∶10. Eight hours after infection at 35°C in an atmosphere containing 5% CO_2_, extracellular parasites were removed by washing with serum-free medium. Infected macrophage cultures were maintained in RPMI 1640 medium plus 10% HIFBS at 37°C with 5% CO_2_ at different drug concentrations. After 72 h, macrophages were fixed for 30 min at 4°C with 2.5% paraformaldehyde in phosphate-buffered saline (PBS; 1.2 mM KH_2_PO_4_, 8.1 mM Na_2_HPO_4_, 130 mM NaCl, and 2.6 mM KCl adjusted to pH 7), and permeabilized with 0.1% Triton X-100 in PBS for 30 min. Intracellular parasites were detected by nuclear staining with Prolong Gold antifade reagent plus DAPI. Drug activity was determined from the percentage of infected cells and the number of amastigotes per cell in drug-treated versus non-treated cultures [17].

### Determination of intracellular levels of non-protein thiols

The levels of non-protein thiols were measured by flow cytometry using CellTracker, as described previously [18]. Parasites (10^7^ promastigotes/mL), grown in M199 medium plus 10% HIFBS were washed twice with PBS and incubated with 2 µM CellTracker for 15 min at 37°C. They were then washed again with PBS and analysed by flow cytometry in a FACScan flow cytometer (Becton-Dickinson, San Jose, CA) equipped with an argon laser operating at 488 nm. Fluorescence emission between 515 and 545 nm was quantified using the Cell Quest software. Non-protein thiol-depleted parasites obtained after incubation with 3 mM BSO (a γ-glutamylcysteine synthetase inhibitor) for 48 h at 28°C were used as controls.

### ATP measurements

ATP was measured using a CellTiter-Glo luminescence assay, which generates a luminescent signal proportional to the amount of ATP present, as described previously [19]. Promastigotes (4×10^6^/mL) were incubated at 28°C in RPMI plus 20% HIFBS containing 0.2 µM AmB or 25 µM MLF for 3 h, or 2 mM Sb^III^ for 8 h. The drug concentration and incubation time were selected by monitoring parasite viability under a microscope. A 25-µL aliquot of parasites was then transferred to a 96-well plate, mixed with the same volume of CellTiter-Glo, and incubated in the dark for 10 min. The resulting bioluminescence was measured using an Infinite F200 microplate reader (Tecan Austria GmbH, Austria).

### Measurement of mitochondrial membrane potential (ΔΨm)

ΔΨm was measured by flow cytometry using Rh123 accumulation, as described previously [20]. The parasites (4×10^6^ promastigotes/mL) were incubated with the drugs as described above. 0.5 µM Rh123 was then added and the parasites incubated for a further 10 min. They were then washed twice, resuspended in PBS and analysed by flow cytometry in a FACScan flow cytometer (Becton-Dickinson, San Jose, CA) equipped with an argon laser operating at 488 nm. Fluorescence emission between 515 and 545 nm was quantified using the Cell Quest software. Parasites fully depolarized by incubation in 10 µM FCCP for 10 min at 28°C were used as controls.

### Statistical analysis

Statistical comparisons between groups were performed using Student's *t*-test. Differences were considered significant at a level of *p*<0.05.

## Results

### Generation of singly and drug combination resistant promastigote lines

The resistant lines were selected *in vitro* in *L. donovani* promastigotes by a stepwise adaptation process, with drug concentrations starting below the EC_50_ values and gradually increasing, over 10 weeks (equivalent to 90 generations), to a maximum concentration of 0.1, 8, 20 and 80 µM for AmB, MLF, PMM and Sb^III^, respectively. Resistance to single drugs and to double drug combinations was induced. The singly AmB-resistant line (A) and the lines resistant to the combination of AmB with MLF (AM), PMM (AP) or Sb^III^ (AS) showed similar EC_50_ values for AmB of 0.14 µM, a value two-fold higher than for the WT line (Table 1). In contrast, the singly MLF-resistant line (M) and the lines resistant to the combination of MLF with AmB (AM) or PMM (MP) showed EC_50_ values for MLF 1.81, 3.10, and 4.43-fold higher than for the WT line, respectively (Table 1). Likewise, the singly PMM-resistant line (P) and the lines resistant to combinations with AmB (AP), MLF (MP) or Sb^III^ (SP), showed EC_50_ values for PMM 11.02, 2.12, 14.25, and 18.35-fold higher than for the WT line, respectively (Table 1). Finally, the line resistant to Sb^III^ alone (S) and the lines resistant to Sb^III^ in combination with AmB (AS) or PMM (SP) showed EC_50_ values for Sb^III^ that were 3.04, 2.05, and 2.18-fold higher than for the WT line, respectively (Table 1). All the resistant lines showed a similar growth rate, morphology, motility and macrophage infectivity to the WT line (data not shown).

**Table 1:** Drugs sensitivity profile in *L. donovani* promastigote lines.

Lines	Drugs EC_50_ (µM)-Resistance Index			
	AmB	MLF	PMM	Sb^III^
**WT**	0.07±0.01	5.84±0.43	12.09±1.76	87.33±5.72
**A**	0.14±0.04^*^ (2.00±0.57)	5.28±0.58 (0.90±0.16)	19.67±1.30^****^ (1.63±0.34)	74.38±4.98^*^ (0.85±0.12)
**M**	0.07±0.03 (1.00±0.56)	10.56±1.06^****,a^ (1.81±0.31)	6.93±0.80 (0.57±0.15)	92.23±7.09 (1.06±0.16)
**P**	0.07±0.02 (1.00±0.42)	4.98±0.38^*^ (0.85±0.12)	133.29±12.36^****,b,c^ (11.02±1.63)	138.15±15.54^****^ (1.58±0.29)
**S**	0.07±0.02 (1.00±0.42)	4.25±0.44^***^ (0.73±0.13)	109.34±2.94^****^ (9.04±0.85)	266.07±10.12^****,d,e^ (3.04±0.32)
**AM**	0.14±0.04^*^ (2.00±0.86)	18.03±1.07^****^ (3.09±0.40)	19.35±3.67^*^ (1.60±0.53)	108.97±4.67^****^ (1.25±0.14)
**AP**	0.14±0.04^*^ (2.00±0.86)	10.86±1.67^****^ (1.86±0.42)	25.67±2.26^****^ (2.12±0.40)	96.69±5.60 (1.11±0.14)
**AS**	0.14±0.04^*^ (2.00±0.86)	5.79±0.40 (0.99±0.13)	22.82±2.92^****^ (1.89±0.51)	178.88±5.91^****^ (2.05±0.49)
**MP**	0.07±0.03 (1.00±0.56)	25.90±1.74^****^ (4.43±0.61)	172.27±3.52^****^ (14.25±1.35)	74.59±3.85^**^ (0.85±0.10)
**SP**	0.07±0.02 (1.00±0.42)	4.61±1.02 (0.79±0.23)	221.88±30.15^****^ (18.35±2.15)	190.57±20.96^****^ (2.18±0.39)

Promastigote forms of *L. donovani* lines: wild-type (WT) and resistant lines to AmB (A), MLF (M), PMM (P), Sb^III^ (S), AmB+MLF (AM), AmB+PMM (AP), AmB+Sb^III^ (AS), MLF+PMM (MP), and Sb^III^+PMM (SP), were grown as described in Materials and Methods section for 72 h at 28°C in the presence of increasing concentrations of drugs. Cell viability was determined using an MTT-based assay. Resistance indexes, between parentheses, were calculated by dividing the EC_50_ for each resistant line by that for WT line. Data are means of EC_50_ or resistance indexes values ± SD from three independent experiments. Significant differences versus the WT were determined using Student's *t*-test (^*^: *p*<0.05; ^**^: *p*<0.01; ^***^: *p*<0.005; ^****^: *p*<0.001) and for comparison of singly versus doubly-resistant lines (^a^: *p*<0.001 M vs. AM or MP; ^b^: *p*<0.05 P vs. MP or SP; ^c^: *p*<0.001 P vs. AP;^ d^: *p*<0.05 S vs. SP; ^e^: *p*<0.005 S vs. AS).

### Maintenance of drug resistance in intracellular amastigotes

We undertook additional experiments to determine whether the resistance to single drugs and drug combinations shown by the promastigote forms was maintained in intracellular amastigotes obtained after infection of mouse peritoneal macrophages (Table 2). The results indicated that the resistance indices to drugs in the different resistant intracellular amastigote lines were maintained and were very similar to those observed in their promastigote counterparts (Tables 1 and 2). The exception was the S-resistant line, which showed a significantly higher resistance index to Sb^III^ (12-fold, Table 2) than that observed for the promastigote form (3-fold, Table 1). The higher resistance index for PMM in the singly P-resistant line (11-fold) and in the MP-(11- and 14-fold for intracellular amastigotes and promastigotes, respectively) and SP- resistant lines (16- and 18-fold for intracellular amastigotes and promastigotes, respectively) it worthy of note. Furthermore, a comparison of AP with P, and AS or SP with S, shows that the doubly resistant lines exhibit lower EC_50_ values for PMM or Sb^III^ than their singly resistant counterparts. In contrast, the SP line exhibits a higher EC_50_ value for PMM than the P line (Table 2).

**Table 2:** Drugs sensitivity profile in *L. donovani* intracellular amastigote lines.

Lines	Drugs EC_50_ (µM)-Resistance Index			
	AmB	MLF	PMM	Sb^III^
**WT**	0.027±0.002	2.18±0.43	3.93±0.60	3.66±0.14
**A**	0.057±0.003^****,a^ (2.110±0.270)	2.03±0.33 (0.93±0.13)	4.43±0.45 (1.13±0.28)	2.38±0.19^***^ (0.65±0.07)
**M**	0.028±0.002 (1.037±0.145)	5.12±0.51^***^ (2.35±0.69)	5.26±0.35 (1.34±0.29)	5.11±0.46^*^ (1.40±0.18)
**P**	0.026±0.003 (0.963±0.180)	1.98±0.23 (0.91±0.28)	41.45±3.78^****,b,c^ (10.55±1.57)	4.66±0.35^*^ (1.27±0.14)
**S**	0.024±0.002 (0.889±0.140)	2.08±0.17 (0.95±0.26)	4.36±0.53 (1.11±0.30)	44.47±4.23^****,d,e^ (12.15±0.92)
**AM**	0.064±0.002^****^ (2.370±0.251)	4.62±0.39^**^ (2.12±0.87)	3.45±0.49 (0.88±0.26)	3.36±0.23 (0.92±0.09)
**AP**	0.058±0.003^****^ (2.148±0.273)	3.98±0.41^*^ (1.83±0.24)	7.10±0.62^****^ (1.81±0.43)	3.98±0.29 (1.09±0.09)
**AS**	0.042±0.003^**^ (1.556±0.342)	3.46±0.54 (1.59±0.16)	3.93±0.51 (1.00±0.28)	6.61±0.57^***^ (1.81±0.22)
**MP**	0.036±0.004 (1.333±0.256)	7.09±1.07^***^ (3.25±1.31)	41.58±3.44^****^ (10.58±1.49)	3.48±0.27 (0.95±0.14)
**SP**	0.026±0.004 (0.963±0.226)	2.46±0.47 (1.13±0.23)	64.48±5.34^****^ (16.41±1.84)	14.05±2.04^***^ (3.84±0.69)

Intracellular amastigote *L. donovani* lines were obtained after infection of mouse peritoneal macrophages with the different promastigote resistant lines as described in Material and Methods section. After 72 h, drug activity was determined from the percentage of infected cells and the number of amastigotes by cells in drug-treated cultures versus non-treated cultures. Resistance indexes, between parentheses, were determined as previously described in Table 1. Data are means of EC_50_ or resistance indexes values ± SD from three independent experiments. Significant differences versus the WT were determined using Student's *t*-test (^*^: *p*<0.05; ^**^: *p*<0.01; ^***^: *p*<0.005; ^****^: *p*<0.001) and for comparison of singly versus doubly-resistant lines (^a^: *p*<0.05 A vs. AS; ^b^: *p*<0.05 P vs. SP; ^c^: *p*<0.001 P vs. AP;^ d^: *p*<0.005 S vs. SP; ^e^: *p*<0.001 S vs. AS).

### Stability of resistance to drug combinations

**Figure 1:**
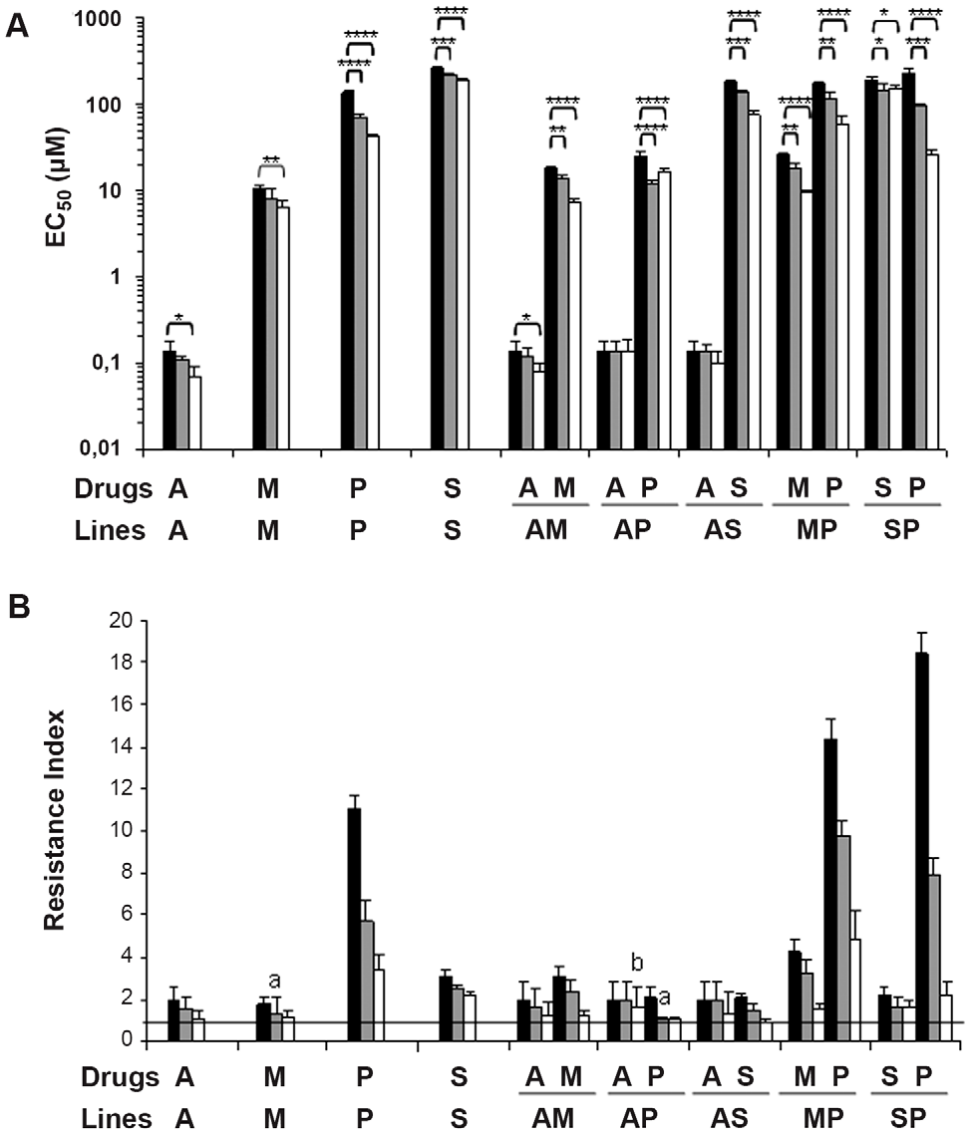
Stability of resistance to drugs in *L. donovani* promastigote lines. (**A**) The stability of resistance with respect to the initial EC_50_ (black columns) is determined at 1 (gray columns) and 4 months (white columns) after removal of the drug pressure. The results shown are the average of three independent experiments ± SD. Significant differences versus the initial EC_50_ were determined using Student's *t*-test (*: *p*<0.05; **: *p*<0.01; ***: *p*<0.005; ****: *p*<0.001). (**B**) Results from panel A expressed in terms of the resistance index. The horizontal black line represents a resistance index of 1 equivalent to a completely loss of resistance. ^a^: line that lose resistance after 1 month in a drug-free medium; ^b^: line that maintain similar initial resistance levels after 4 months in a drug-free medium.

The resistant phenotypes were stable in a drug-free medium for 1 month in the singly A-resistant and AM, AP, and AS doubly-resistant lines for AmB. In contrast, the remaining resistances were unstable, although the EC_50_ values were higher than for the WT line, except for the M and AP lines, which lost resistance against MLF and PMM, respectively (Figure 1). After culture for four months in a drug-free medium, all lines lost their resistance levels either completely or partially, except the AP line, which maintained a similar initial resistance level for AmB (Figure 1). These findings suggest that the resistance phenotype of the induced drug combination resistance is unstable.

### Cross-resistance profile to drugs in resistant lines

**Figure 2:**
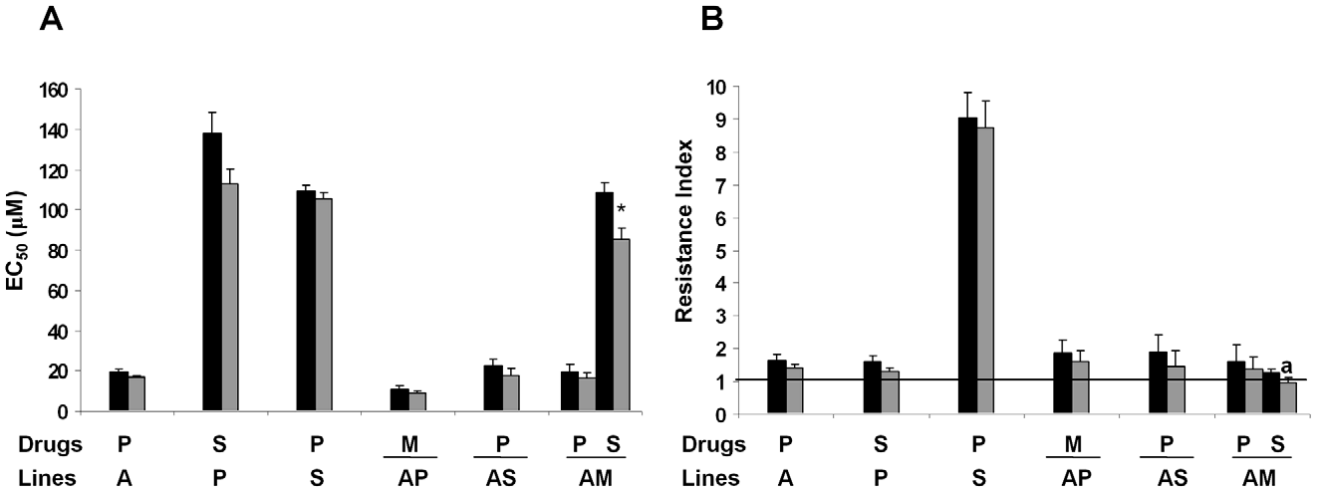
Stability of cross-resistance to drugs in *L. donovani* promastigote lines. (**A**) The stability of cross-resistance at 1 month after removal of drug pressure (gray columns), relative to initial EC_50_ (black columns), was determined. The results are the average of three independent experiments ± SD. Significant differences versus the initial EC_50_ were determined using Student's *t*-test (*: *p*<0.005). (**B**) Results from panel A expressed in terms of resistance index. The horizontal black line represents a resistance index of 1 equivalent to a completely loss of resistance. ^a^: line that lose resistance after 1 month in a drug-free medium.

We investigated the cross-resistance profile of the promastigote and intracellular amastigote forms of each resistant line to different anti-leishmanial drugs (Tables 1 and 2). Both the A- and S-resistant lines showed a significant cross-resistance profile to PMM (Table 1), and the P-resistant line showed resistance to Sb^III^ (Table 1). However, the M and P lines only showed resistance to Sb^III^ in their intracellular amastigote forms (Table 2). In the case of drug combination resistant lines, we found that the AP-resistant line showed significant cross-resistance to MLF in both its promastigote and intracellular amastigote forms. Similarly, the AS-resistant promastigote line shows cross-resistance to PMM, and the AM-resistant promastigote line shows cross-resistance to PMM and Sb^III^ (Tables 1 and 2). However, the MP and SP lines showed no cross-resistance to other anti-leishmanial drugs as either promastigotes or intracellular amastigotes (Tables 1 and 2). Our results concerning the stability of the cross-resistance in resistant lines maintained without drug pressure for one month showed that all lines maintained their resistance, except the AM line, which lost its cross-resistance to Sb^III^ (Figure 2).

### Intracellular thiol levels in resistant lines

**Figure 3:**
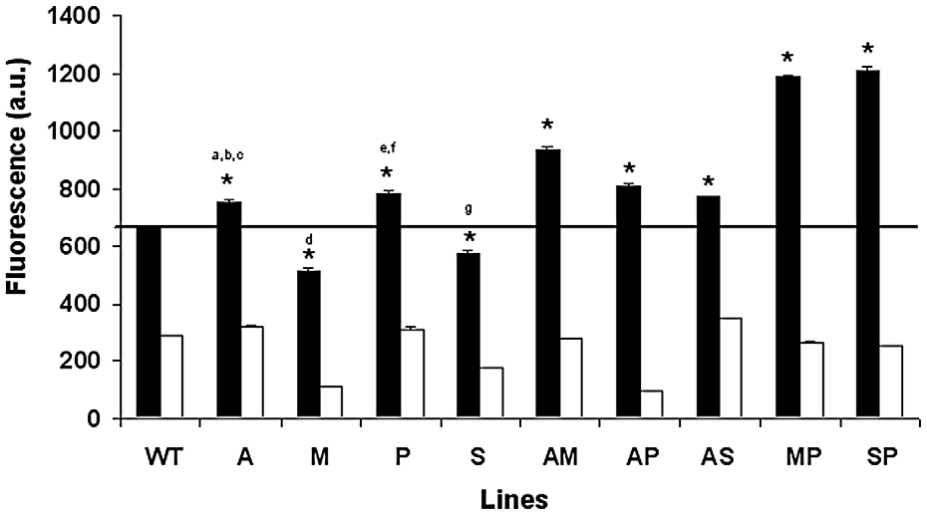
Thiol levels in *L. donovani* promastigote lines. Log-phase promastigotes from WT and resistant lines were labelled with 2 µM CellTracker (black columns), and fluorescence was analyzed as described in the Materials and Methods section. Promastigotes were depleted of thiols by treatment with 3 mM BSO (a γ-glutamylcysteine synthetase inhibitor) for 48 h at 28°C (white columns). The results are the average of three independent experiments ± SD. All lines show significant differences with respect to WT (*: *p*<0.001) and when comparing singly versus doubly-resistant lines using Student's *t*-test (^a^: *p*<0.05 A vs. AS; ^b^: *p*<0.005 A vs. AP; ^c^: *p*<0.001 A vs. AM; ^d^: *p*<0.001 M vs. AM or MP; ^e^: *p*<0.05 P vs. AP; ^f^: *p*<0.001 P vs. MP or SP; ^g^: *p*<0.001 S vs. AS or SP). The black line indicates the threshold at which the thiol levels have been modified with respect to WT.

An increase in thiol levels has been considered to be one of the main detoxification mechanisms observed in lines selected for resistance to Sb^III^ [21]. In light of this, we determined the total intracellular non-protein thiol content in the different resistant promastigote lines using CellTracker. The results of this study showed significantly higher thiol levels in the resistant lines than in the WT line, except for the M and S lines (Figure 3). The highest thiol values were found in the MP and SP lines. As expected, a drastic decrease in thiol content was observed in all lines after incubation with BSO (Figure 3).

### The effect of anti-leishmanial drugs on ATP synthesis in drug-resistant lines

**Figure 4:**
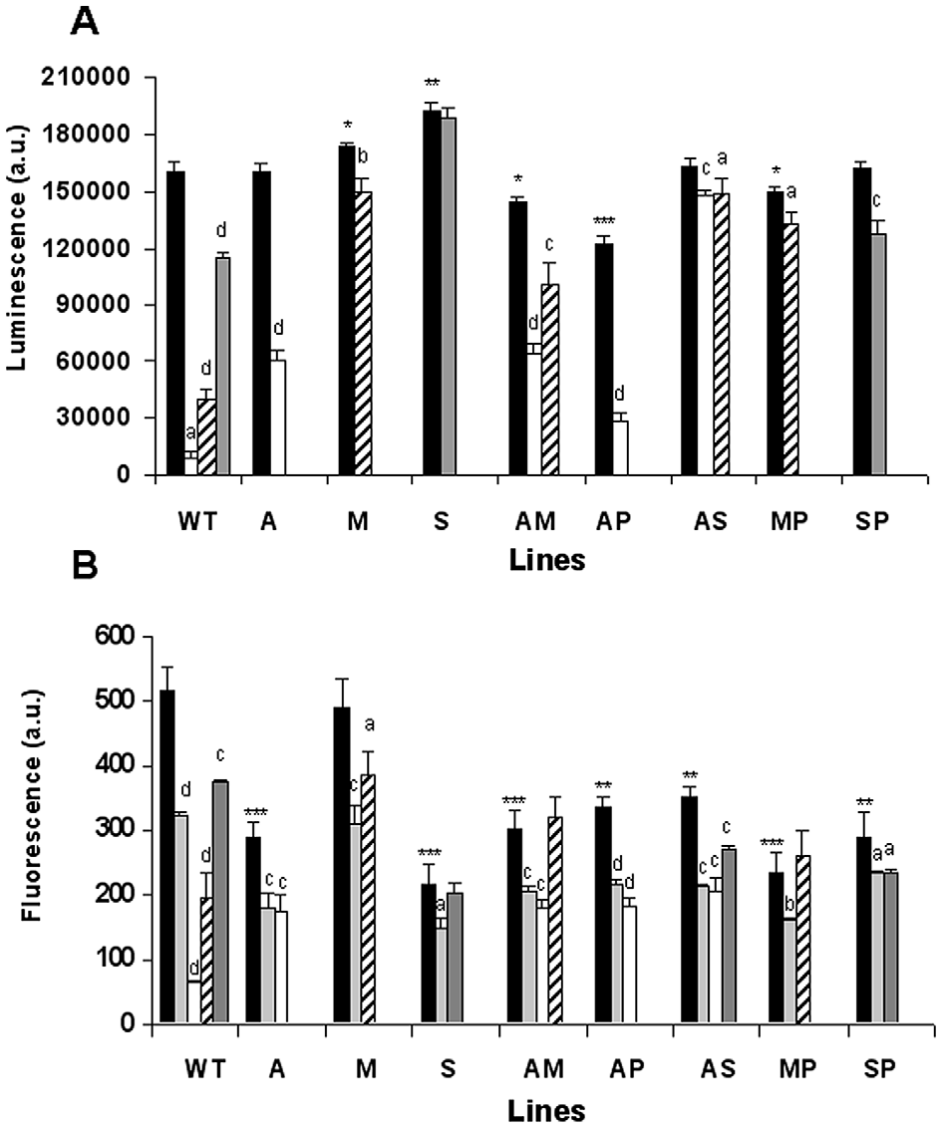
Effect of anti-leishmanial drugs on ATP synthesis and ΔΨm in resistant *L. donovani* promastigote lines. (**A**) ATP levels were measured using CellTiter-Glo. (**B**) ΔΨm was measured by determining the accumulation of Rh123 (0.5 µM) in WT and resistant lines. In both cases, promastigote log-phase cultures were left untreated (controls, black columns) or exposed to 0.2 µM AmB (white columns), 25 µM MLF (oblique lines columns) for 3 h, 2 mM Sb^III^ (gray columns) for 8 h, or 10 µM FCCP as a depolarization control (light gray columns) for 10 min. Measurements are expressed in arbitrary luminescence (panel A) or fluorescence (panel B) units ± SD from three independent experiments and show significant differences (^a^: *p*<0.05; ^b^: *p*<0.01; ^c^: *p*<0.005; ^d^: *p*<0.001) when comparing the corresponding control values for each line with itself after treatment. A comparison of the different untreated lines with untreated WT lines also shows significant differences (*: *p*<0.05; **: *p*<0.005; ***: *p*<0.001).

We assessed the ATP levels in the presence of AmB, MLF and Sb^III^, which are known to induce an apoptotic-like process associated with ATP depletion in *Leishmania* [22]. PMM was not assessed as this drug kills the parasites by a different mechanism [22]–[24]. The WT parasites exhibited a significant decrease in total ATP levels after treatment with AmB, MLF or Sb^III^, with the former showing the highest decrease (Figure 4A). The lines resistant to single and drug combinations remained protected or were more tolerant to ATP loss (Figure 4A), with the AS-resistant line in particular showing a very small decrease in ATP levels after treatment with AmB or Sb^III^. A similarly small decrease in ATP levels was also observed after treatment of the M- and MP-resistant lines with MLF, and the S-resistant line with Sb^III^ (Figure 4A). Additionally, we observed that the M- and S-resistant lines presented significantly higher basal ATP levels without drug pressure (Figure 4A), thus suggesting that these resistant lines have developed, amongst other resistance mechanisms, an increase in ATP levels. In contrast, the AM- and AP-resistant lines presented significantly lower basal ATP levels (Figure 4A).

### Tolerance to drug-induced depolarization of ΔΨm in drug-resistant lines

ΔΨm is essential to mitochondrial ATP synthesis and changes to it are one of the markers for apoptosis induced by exposure to AmB, MLF and Sb^III^ (but not exposure to PMM) [22]. Furthermore, mitochondrial oxidative phosphorylation in *Leishmania* accounts for most of the ATP expenditure of *Leishmania* parasites [25]. As a result, we tested the ΔΨm of WT and the various resistant lines by measuring Rh123 accumulation (Figure 4B). WT parasites incubated with AmB, MLF or Sb^III^ showed a significant decrease in Rh123 accumulation (7.7-, 2.6- and 1.4-fold, respectively). These values (except for that for Sb^III^) were even lower than those obtained upon incubation of parasites with the control uncoupling reagent FCCP (Figure 4B). Except for the M-resistant line, the untreated resistant lines showed a significantly lower accumulation of Rh123; however, after treatment with the different anti-leishmanial drugs, the resistant lines showed a lower reduction ratio of Rh123 accumulation than the WT line (Figure 4B). Consequently, the resistant parasites remain protected against the oxidative stress induced by treatment with the anti-leishmanial drugs AmB, MLF and Sb^III^.

## Discussion

Drug combinations for the treatment of leishmaniasis represent a promising and challenging chemotherapeutic strategy that has recently been implemented in different endemic areas. This approach has several advantages over single-drug therapies, including shortening of the treatment period and reduction of the probability of selecting drug-resistant parasites. However, this approach must be used with care given to the possibility that, if not applied in a controlled and regulated way, resistance could be induced in *Leishmania*, thus resulting in a rapid loss of efficacy of not one but two therapeutic options [13]. It is therefore important to design relevant experimental studies in order to determine whether *Leishmania* parasites are able to develop resistance to the different potential anti-leishmanial drug combinations that are likely to be used in the near future. The results obtained from such experimental studies could help to predict the likely success of drug combination therapy.

There is still a great deal of debate concerning the clinical relevance of findings in promastigotes since this is not the stage that will eventually become exposed to the drug. It has recently been shown that differences can be obtained during the experimental induction of resistance to PMM using promastigotes and intracellular amastigotes depending on the resistance-selection protocol [26]. The methodology and technical difficulties required in inducing resistance to drug combinations justify the use of promastigote forms in this manuscript. However, experiments using intracellular amastigotes derived from resistant promastigotes could be useful when considering future recommendations for optimal drug combinations to combat different *Leishmania* species.

Studies in clinically resistant isolates, where the mechanisms of resistance involve multi-factorial events that contribute to the tolerance to chemotherapeutic agents in *Leishmania*, are somewhat more complex [27], [28]. In this paper, we have induced experimental resistance to the drug combinations AM, AP, AS, MP and SP in *L. donovani* (MHOM/ET/67/HU3, also known as LV9 or L82). The sensitivity values of the parental *L. donovani* strain to the different anti-leishmanial drugs used were similar to, or even lower than, the published data for this and other *L. donovani* strains [11], [26], [29]–[31]. It is important to point out that this is the first description of an experimental induction of resistance to a combination of different anti-leishmanial drugs. Consequently, the conditions and times required for the induction of resistance can not be compared to the previously described induction of resistance to single anti-leishmanial drugs. Additionally, the single-drug resistance studies in *L. donovani* described by different groups, obtained higher levels of resistance after increasing the drug pressure and exposure times. Thus, an approximately 14-fold resistance has been obtained for MLF resistance [32], an approximately 5- to11-fold resistance for PMM, depending on the *L. donovani* strain used [26], and an approximately 20-fold resistance for AmB [31]. Moreover, it is important to note that, in clinical isolates resistant to sodium stibogluconate, an up to 41-fold higher tolerance to Sb^III^ has been observed with respect to the susceptible clones of promastigote forms in the stationary growth phase [27].

In summary, the results of this study show that *L. donovani* can easily develop resistance to the drug combinations MLF/PMM and Sb^III^/PMM, with higher resistance indices than those found for AmB/MLF, AmB/PMM or AmB/Sb^III^. These results have been validated in intracellular amastigotes and are of considerable interest for future applications. Experimental resistance of *L. donovani* to the drug combination MLF/PMM, a combination that could, in theory, have advantages over other drug combinations as regards future use, is easily achieved. Similarly, the experimental studies described herein confirm how the ease with which experimental resistance to Sb^III^/PMM is induced in *Leishmania*. These studies should therefore be taken into account when it comes to future recommendations for their use in endemic areas, especially as the Sb^III^/PMM combination appears to be effective against VL (in East Africa) and has the additional advantage of low cost [7]. Consequently, further research into combination regimens when given for a short period and at lower total dose are required.

We have also confirmed that *Leishmania*-resistant parasites develop an increase in cellular thiol redox metabolism as a drug-detoxification mechanism to protect against drug-induced loss of ATP and mitochondrial membrane potential. As described previously, the anti-leishmanial drugs AmB, MLF and Sb^III^ (but not PMM) induce a significant decrease in the mitochondrial membrane potential, thus leading to a bioenergetic collapse of the parasite and drug-induced cell-death [22]. A link between the mode of killing of drugs against *Leishmania infantum* (such as AmB, MLF and Sb, which share a similar mode of killing), and the tolerance towards cell death induced by their respective anti-leishmanial drugs, has been described previously [22]. Although, in contrast to PMM, this was thought to facilitate the emergence of multidrug resistance, similar findings were not observed under our experimental conditions. Instead, our results show a significant cross-resistance profile to PMM in the AM- and AS-resistant lines and to MLF in the AP-resistant line. Conversely, the AP-resistant intracellular amastigote line acquired cross-resistance to MLF. A similar absence of a link between cross-resistance to drugs with similar mechanism-of-death pathways was observed in the singly A- and S-resistant promastigote lines, with a cross-resistance to PMM, and in the P-resistant line, with resistance to Sb^III^. The absence of any such correlation could be explained by taking into account that different *Leishmania* species present different drug susceptibilities and different abilities to respond to drug pressure.

In light of the characteristics of this infectious disease and the existence of different *Leishmania* species, with their different drug susceptibilities, it is possible that each *Leishmania* species will require a different drug combination. Suitable options for combination treatment must therefore be optimised in further experimental studies. In this respect, genome-sequencing and metabolomics experiments are currently underway to determine the specific resistance mechanisms developed by *Leishmania* parasites to different drug combinations. Finally, in view of the proven value of these results for the research community, and considering the debate as regards the use of promastigotes or intracellular amastigotes for induction of drug resistance to drug combinations, we are currently attempting to induce resistance to drug combinations in intracellular amastigotes, as the results obtained will be of greater significance in terms of the conditions found in the field.
